# Cardiovascular disease in pregnancy: the South African perspective

**Published:** 2016

**Authors:** John Anthony, Karen Sliwa, Andrew Sarkin

**Affiliations:** Division of Obstetrics and Gynaecology, Groote Schuur Hospital, University of Cape Town, South Africa; Hatter Institute for Cardiovascular Research in Africa, and IDM, Department of Medicine, Faculty of Health Sciences, University of Cape Town, South Africa; Soweto Cardiovascular Research Unit, University of the Witwatersrand, Johannesburg; Inter-Cape Heart Group, Medical Research Council South Africa, Cape Town, South Africa; Department of Cardiology, Faculty of Health Sciences, University of Pretoria, South Africa

Maternal mortality in South Africa, as in many developing nations, is avoidably high. The causes of death are well documented because statutory notification of mortality, happening during pregnancy and for 42 days after delivery, has been in place for 15 years now. The mortality data have been compiled into a triennial report (Saving Mothers) published by the National Department of Health.^1^ These reports map the epidemiology of avoidable maternal mortality, for which there are diverse causes, none more significant than the dual failure on the part of attending clinicians to correctly identify potentially life-threatening illness, together with recurrent failure to provide an adequate standard of care to ill pregnant women.

The death of a pregnant woman may adversely affect the chance that her surviving children will thrive. In South Africa approximately 1 600 women die every year because of pregnancy complications. Many others suffer the burden of on-going morbidity related to childbirth. Preventing premature death and disability among women and children is a priority to which the National Department of Health has committed itself. Given the pivotal role of women in society, especially within poorer communities, this targeted intervention is one with which few would take issue.

The epidemiology of maternal mortality informs a variety of proposed recommendations aimed at reducing the risk of death related to childbirth. The burden of disease is described by Soma-Pillay and Sliwa in this issue (page 60). The contribution of cardiac disease in pregnancy is recognised to be the single most prevalent medical disorder giving rise to death during pregnancy among South African women. Reducing deaths due to cardiac disease has not yet been accomplished. The need for accurate diagnosis and appropriate management depends on identifying women with some evidence of cardiac disease, followed by referral to an appropriate level of medical care where the greatest available level of expertise may be employed in the further management of such patients.

However, such a simple principle is difficult to implement. Often those providing care at the community level (where most South African women deliver their babies) are ill-equipped to recognise significant disease and even less able to provide the necessary medical management. Innovative approaches have been necessary and are also part of the recommendations made in the triennial report.

Sliwa *et al.* have described the function of a combined obstetric and cardiac clinic where multi-disciplinary care is provided to women with suspected heart disease.^2^ The object of this clinic is to diagnose, triage and implement care during pregnancy and to ensure that those who present with undiagnosed disease during pregnancy have ongoing access to care after childbirth. Preconception counselling and contraceptive advice are all provided within the same clinical environment. The triennial report has endorsed this type of combined clinic that encompasses the skills of both obstetricians and cardiologists as a means to eliminate any failure to recognise problems correctly and to ensure that the incidence of substandard care is kept to a minimum.

Such clinics are feasible in metropolitan areas of the country where the greatest concentration of people live. Smaller towns and rural communities have less access to the same level of care. Nevertheless, co-responsibility for patient care between practitioners with different skills sets is recognised to be beneficial, and combined obstetric and medical clinics have been suggested as an attainable goal throughout the country. Monthly joint clinics would enable more considered evaluation of suspected medical disorders during pregnancy and an enhanced level of care together with appropriate referral to regional hospitals.

The difficulty of discerning between normal pregnancy physiology and clinical disease, as well as understanding the impact of pregnancy physiology on underlying medical disease has not been taught or examined in the post-graduate training curriculum of general physicians. The anticipated benefits of combined care would only be realised once essential aspects of pregnancy physiology and pathophysiology and their influence on the expression and management of medical disease complicating pregnancy is incorporated into the university curriculum. Such changes are under consideration at present and to that end, this publication establishes a template for understanding the epidemiology of cardiac problems in pregnancy, understanding the (patho)physiology of pregnancy and how interventions, both obstetric and medical, may influence the outcome of these pregnancies. The broader object of this process still remains the targets set 15 years ago and enunciated as the millennium development goals.^3^
